# Patient satisfaction following resin‐bonded fixed dental prostheses cemented by using the Dahl concept

**DOI:** 10.1002/cre2.774

**Published:** 2023-08-25

**Authors:** Tong Wah Lim, Rostam Iffendi Idris, Melati Mahmud

**Affiliations:** ^1^ Division of Restorative Dental Sciences, Faculty of Dentistry The University of Hong Kong Pok Fu Lam Hong Kong; ^2^ Kulliyyah of Dentistry International Islamic University Malaysia Kuantan Malaysia; ^3^ Centre of Restorative Dentistry Studies, Faculty of Dentistry Universiti Teknologi MARA Sungai Buloh Malaysia

**Keywords:** patient satisfaction, resin‐bonded fixed dental prosthesis, surveys and questionnaires, vertical dimension

## Abstract

**Objective:**

Resin‐bonded fixed dental prostheses (RBFDPs) cemented at an increased occlusal vertical dimension (OVD) (the Dahl concept) to create space for a metal retainer remains controversial because of the lack of reported clinical studies. This study analyzed the demographic (age and sex) and clinical factors (location and arch of prosthesis) affecting the patients' perception of RBFDPs cemented at an increased OVD.

**Material and Methods:**

Twenty‐eight participants treated with cantilevered RBFDP at an increased OVD were prospectively recruited. They were asked to answer a validated patient satisfaction questionnaire based on six parameters during the 12‐week review visit.

**Results:**

71.4% of the participants were completely satisfied with the color, shape, and function. Twenty‐one (75%) participants reported no complaints about the prostheses. 89.3% will recommend this treatment option to others. There was a significant difference between males and females in avoiding loading on the prostheses (*p* = 0.015). The level of satisfaction did not differ by age, ethnicity, location, and arch of the prostheses (*p* > 0.05).

**Conclusions:**

Patient satisfaction toward RBFDP cemented by using the Dahl approach was generally high on all the parameters at the 12‐week review visit.

## 1 INTRODUCTION

A resin‐bonded fixed dental prosthesis (RBFDP) is a minimally invasive treatment option for the replacement of the missing tooth. Currently, RBFDPs are gaining in popularity because of the preservation of tooth structure, no surgery, moderate to high survival rates, short duration of treatment, and economics (Lim et al., 2014; Thoma et al., 2017). Conventionally, interocclusal space for RBFDP retainer is created after occlusal preparation of the abutment tooth or alteration of the antagonist tooth with the aid of diagnostic wax‐up (Lim et al., 2014). Recently, the application of the Dahl concept (Dahl et al., 1975) has shown a good outcome for interocclusal space creation for the RBFDPs (Idris et al., 2022; Lim, 2022; Lim et al., 2022a). The abutment tooth preparation is limited to the removal of the undercut to ease the path of insertion and maximize the connector and retainer height or no preparation at all (Dayanik, 2016; Lim et al., 2022a). The Dahl concept is defined as “the relative axial tooth movement that is observed when a localized appliance or localized restorations are placed in supra‐occlusion and the occlusion reestablishes full arch contacts over a period of time” (Poyser et al., 2005).

Preservation of the enamel to maximize the bonding, ease of application, short chair‐side time, and elimination of the use of interim restorations are the main advantages of the application of the Dahl concept. The reported clinical complications, particularly partial occlusal reestablishment and widening of periodontal ligament space, may be the main drawbacks of this technique, even though the number was small (Lim et al., 2022a). However, patients with partial occlusal reestablishment were reported to function well as their occlusal force was reestablished after 3 months (Idris et al., 2022). Almost 90% achieved complete occlusal reestablishment after the RBFPDPs had been placed at an increased occlusal vertical dimension (OVD) (Idris et al., 2022; Lim et al., 2022a), whereby the 94%−100% occlusal reestablishment has been found in the treatment of localized tooth wear (Gough & Setchell, 1999; Poyser et al., 2005; Redman et al., 2003).

The treatment outcome of RBFDPs by evaluating patient satisfaction has been identified and examined in a clinical study by Creugers and De Kanter (2000). There were six parameters in relation to the satisfaction toward RBFDPs were evaluated including “overall function,” “color,” “shape,” “functional changes,” “complaints,” and “recommendations to other patients.” The degrees of satisfaction according to the different parameters were high, except for the “color.” Only 68% of the patients scored Alpha (satisfied) during the evaluation of the color scores. However, it was found that 95.2% of patients were happy with the prosthetics' esthetics and with the whole prosthetic experience in a retrospective study in Hong Kong (Botelho et al., 2014). In the same center, Lam et al. (2019) also reported high acceptance of 132 posterior fixed‐movable RBFPDPs after 5 years.

In a cross‐sectional study, the treatment satisfaction between RBFDPs and dental implants was not influenced by gender, age, and site of the prostheses (Lim & Ariff, 2020). However, there was a significantly higher satisfaction score of dental implants in relation to the existing appearance and mastication compared to RBFDPs, supported by the study of Hebel et al. (2000). Most of the dissatisfaction of the patients toward RBFDP was due to the gray shadow or the metal appearance of the retainer on the abutment tooth (Djemal et al., 1999). A comparison between removable partial dentures and distal cantilevered RBFDPs in the treatment of distal extension edentulism was investigated in a randomized control trial (Jepson et al., 2003). Patient satisfaction between both prostheses was compared at baseline, 3 months, and 1 year after the provision of the prostheses. Both groups demonstrated significant improvements in perceived masticatory skills and patient comfort.

In brief, all the previous studies assessed medium to long‐term patient satisfaction after receiving RBFDPs. However, the authors are unaware of a study investigating patient satisfaction after placement of the RBFDPs using the Dahl concept. Therefore, the present study aimed to evaluate patient satisfaction and analyze the demographic (age and sex) and clinical factors (location and arch of prosthesis) affecting the patients' perception of RBFDPs cemented at an increased OVD. Clinicians could benefit from an understanding of the treatment outcome and patient behavior toward RBFDPs. The null hypothesis was that no significant relationship between patient satisfaction after the RBFDP cemented using the Dahl concept with demographic and clinical factors.

## 2 MATERIALS AND METHODS

This was a cross‐sectional study to explore patient satisfaction following RBFDPs cemented at an increased OVD using a self‐administered questionnaire. Different data sets and the same 28 participants were derived from a previously published prospective clinical study (Idris et al., 2022). Ethics clearance was obtained from the Institute of Research Management and Innovation with a reference number of 600‐IRMI (5/6/1) REC/352/17. A convenience sampling method was adopted in this study, participants who complied with the following inclusion criteria were enrolled: a participant treatment planned to receive a cantilevered 2‐unit RBFDP replacing an anterior tooth or a premolar with an interproximal edentulous span of less than 10 mm, and RBFDP was cemented at an increased OVD, a vital abutment tooth with a healthy periodontium, and the presence of at least 10 pairs of natural teeth in occlusal contacts. Participants were excluded if they had a removable partial dental prosthesis or a systemic disease that affected jaw function, developmental anomalies, temporomandibular joint disorder, history of jaw injury, or periodontal disease. Before being enrolled in this study, each participant gave their written informed consent. In this study, no occlusal preparation was performed for the retainer space except for minimal preparation on the axial walls consisting of removing undercuts for the abutment to increase the connector height and facilitate the path of insertion. The RBFDPs were cemented using the Dahl concept, and the increased OVD was established from the thickness of the metal retainers after cementation, which ranged from 0.5 to 1.0 mm. The metal framework of the RBFDPs was fabricated using a nickel‐chrome base metal alloy (Wiron Light; BEGO Bremer Goldschlägerei Wilh. Herbst GmbH & Co KG) with a complete coverage retainer design. A standard cementation protocol for RBFDP was adopted (Idris et al., 2022), and all participants were advised of the possible risks and complications.

The participant's demographic and clinical factors of age, gender, location (anterior or posterior), and arch of the prosthesis (maxillary or mandibular) were documented before the cementation of the RBFDPs. A set of patient satisfaction questionnaires from Creugers and De Kanter (2000) was adopted in the present study. The questionnaires consisted of six types of parameters; (i) “overall function,” “color,” “shape,” “function,” (ii) “functional changes,” “remarks on function,” “complaints,” (iii) para‐functional habits such as “playing,” (iv) “avoidance of loading,” (v) “preference for another type of restoration,” and (vi) “recommendation to other patients.” The participants were called for a 12‐week review visit after the RBFDPs were delivered. They were asked to fill in the questionnaire independently during the review visit. The data were analyzed using a statistical software program (IBM SPSS Statistics; v25.0; IBM Corp.). *χ* ^2^ test was used with a significance value of *p* < 0.05.

## 3 RESULTS

**Figure 1:**
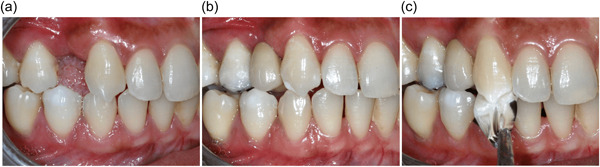
(a) Buccal view of precementation stage showing the missing maxillary right first premolar. (b) Buccal view of post‐cementation stage showing resin‐bonded fixed dental prosthesis cemented at increased occlusal vertical dimension. (c) Buccal view of 12‐week review showing occlusal reestablishment achieved by using shim stock foil.

A total of 28 adult participants (16 females and 12 males) have been recruited for this study. Table 1 presents the demographic and treatment characteristics of the participants. The mean age for participants was 44 years old. Half of the cantilevered RBFDPs are located at the anterior segment. Seventeen (60.7%) of the RBFDPs replaced maxillary missing teeth. As reported in the previous study, a total of 25 (89.3%) of the participants achieved complete occlusal reestablishment compared to 3 (10.7%) were partial occlusal reestablishment after 12 weeks (Idris et al., 2022) (Figure 1).

**Table 1:** Baseline characteristics.

Parameter		Frequency *n* (%)
Age (year)	<39	13 (46.4)
	40−59	12 (42.9)
	>60	3 (10.7)
Sex	Male	12 (42.9)
	Female	16 (57.1)
Location of prosthesis	Anterior	14 (50.0)
	Posterior	14 (50.0)
Arch of prosthesis	Maxilla	17 (60.7)
	Mandible	11 (39.3)

Twenty (71.4%) participants were completely satisfied with the color, shape, and function. Twenty‐one (75.0%) of them reported no complaints about functional changes during the review of RBFDPs. Almost 54% of the participants often avoid loading on the prostheses. Twenty‐four (85.7%) of them declared not to play the RBFDPs with their tongues. There were only 2 (7.1%) participants who unfavoured this type of prosthesis, and most of them (89.3%) reported that they would recommend this treatment option to others (Table 2).

**Table 2:** Characteristics of patient satisfaction.

Patient satisfaction parameters		Frequency (%)
Color, shape, overall function	Reasonably satisfied	8 (28.6)
	Completely satisfied	20 (71.4)
Complaints, remarks on function, functional changes	Not sure	7 (25.0)
	No	21 (75.0)
Avoidance of loading	Often or always	15 (53.6)
	Sometimes or regularly	8 (28.6)
	No	5 (17.9)
Parafunctional habits (playing with tongue)	Sometimes or regularly	4 (14.3)
	No	24 (85.7)
Preference for another type of restoration	Yes	2 (7.1)
	Not sure, do not know	7 (25.0)
	No	19 (67.9)
Recommendations to other patients	Not sure, do not know	3 (10.7)
	Yes	25 (89.3)

Upon Pearson's *χ* ^2^ or Fisher's exact test, most of the demographic (age) and clinical (location and arch of the prostheses) factors were not significantly associated with the patient satisfaction parameters toward RBFDPs (*p* > 0.05) (Tables 3, 4, 5). However, Table 6 shows a significant difference for the males in avoiding loading on RBFDPs compared to the females (*p* = 0.015).

**Table 3:** Relationship between patient satisfaction and age.

		Age (year)			*p* Value
Patient satisfaction parameters *n* (%)		<39	40−59	>60	
Color, shape, overall function	Reasonably satisfied	1 (7.7)	6 (50.0)	1 (33.3)	0.064
	Completely satisfied	12 (92.3)	6 (50.0)	2 (66.7)	
Complaints, remarks on function, functional changes	Not sure	2 (15.4)	4 (33.3)	1 (33.3)	0.550
	No	11 (84.6)	8 (66.7)	2 (66.7)	
Avoidance of loading	Often or always	8 (61.5)	6 (50.0)	1 (33.3)	0.188
	Sometimes or regularly	4 (30.8)	4 (33.3)	0	
	No	1 (7.7)	2 (16.7)	2 (66.7)	
Parafunctional habits (playing with tongue)	Sometimes or regularly	1 (7.7)	2 (16.7)	1 (66.7)	0.495
	No	12 (92.3)	10 (83.3)	2 (66.7)	
Preference for another type of restoration	Yes	1 (7.7)	1 (8.3)	0	0.986
	Not sure, do not know	3 (23.1)	3 (25.0)	1 (33.3)	
	No	9 (69.2)	8 (66.7)	2 (66.7)	
Recommendations to other patients	Not sure, do not know	1 (7.7)	1 (8.3)	1 (33.3)	0.407
	Yes	12 (92.3)	11 (91.7)	2 (66.7)	

**Table 4:** Relationship between patient satisfaction and location of prosthesis.

		Location of prosthesis		*p* Value
Patient satisfaction parameters *n* (%)		Anterior	Posterior	
Color, shape, overall function	Reasonably satisfied	5 (35.7)	3 (21.4)	0.678
	Completely satisfied	9 (64.3)	11 (78.6)	
Complaints, remarks on function, functional changes	Not sure	4 (28.6)	3 (21.4)	1.000
	No	10 (71.4)	11 (78.6)	
Avoidance of loading	Often or always	7 (50.0)	8 (57.1)	0.145
	Sometimes or regularly	6 (42.9)	2 (14.3)	
	No	1 (7.1)	4 (28.6)	
Parafunctional habits (playing with tongue)	Sometimes or regularly	2 (14.3)	2 (14.3)	1.000
	No	12 (85.7)	12 (85.7)	
Preference for another type of restoration	Yes	1 (7.1)	1 (7.1)	0.907
	Not sure, do not know	3 (21.4)	4 (28.6)	
	No	10 (71.4)	9 (64.3)	
Recommendations to other patients	Not sure, do not know	1 (7.1)	2 (14.3)	1.000
	Yes	13 (92.9)	12 (85.7)	

**Table 5:** Relationship between patient satisfaction and arch of prosthesis.

		Arch of prosthesis		*p* Value
Patient satisfaction parameters *n* (%)		Maxilla	Mandible	
Color, shape, overall function	Reasonably satisfied	5 (29.4)	3 (27.3)	1.000
	Completely satisfied	12 (70.6)	8 (72.7)	
Complaints, remarks on function, functional changes	Not sure	5 (29.4)	2 (18.2)	0.668
	No	12 (70.6)	9 (81.8)	
Avoidance of loading	Often or always	10 (58.8)	5 (45.5)	0.112
	Sometimes or regularly	6 (35.3)	2 (18.2)	
	No	1 (5.9)	4 (36.4)	
Parafunctional habits (playing with tongue)	Sometimes or regularly	3 (17.6)	2 (9.1)	1.000
	No	14 (82.4)	12 (90.9)	
Preference for another type of restoration	Yes	1 (5.9)	1 (9.1)	0.780
	Not sure, do not know	5 (29.4)	2 (18.2)	
	No	11 (64.7)	8 (72.7)	
Recommendations to other patients	Not sure, do not know	2 (11.8)	1 (9.1)	1.000
	Yes	15 (88.2)	10 (90.9)	

**Table 6:** Relationship between patient satisfaction and sex.

		Sex		*p* Value
Patient satisfaction parameters *n* (%)		Male	Female	
Color, shape, overall function	Reasonably satisfied	3 (25.0)	5 (31.3)	1.000
	Completely satisfied	9 (75.0)	11 (68.8)	
Complaints, remarks on function, functional changes	Not sure	2 (16.7)	5 (31.3)	0.662
	No	10 (83.3)	11 (68.8)	
Avoidance of loading	Often or always	9 (75.0)	6 (37.5)	0.015*
	Sometimes or regularly	0 (0.0)	8 (50.0)	
	No	3 (25.0)	2 (12.5)	
Parafunctional habits (playing with tongue)	Sometimes or regularly	1 (8.3)	3 (18.8)	0.613
	No	11 (91.7)	13 (81.3)	
Preference for another type of restoration	Yes	0 (0.00	2 (12.5)	0.437
	Not sure, do not know	3 (25.0)	4 (25.0)	
	No	9 (75.0)	10 (62.5)	
Recommendations to other patients	Not sure, do not know	1 (8.3)	2 (12.5)	1.000
	Yes	11 (91.7)	14 (87.5)	

* Significant at *p* < 0.05, *χ* ^2^ test.

## 4 DISCUSSION

This study aimed to evaluate patient satisfaction after placement of the RBFDPs using the Dahl concept. There was a significant difference between the males and females in avoiding loading on the prostheses, thus partially rejecting the null hypothesis. The present cross‐sectional study outcome was directly related to the RBFDPs and explored its relationship with the demographic and clinical factors. Therefore, no baseline data and comparison with other treatments can be made. The metal retainer space creation technique in this study, which was the placement of the RBFDPs using the Dahl concept, may be a concern for some clinicians regarding postoperative complications triggered by changes in the masticatory system and poor patient adaptation. Furthermore, a review reported that the implications of this approach may cause pain and discomfort (Moreno‐Hay & Okeson, 2015). However, some studies have reported contradictory results in relation to the safety and effectiveness of raising OVD in restorative treatments (Abduo & Lyons, 2012; Lim, 2022; Lim et al., 2022b; Ormianer & Gross, 1998).

The high degree of patient satisfaction in this study may be explained by the no or minimal tooth preparation required for the RBFDPs cemented at an increased OVD. Less invasive treatment has been shown to have high patient satisfaction (Malmstrom et al., 2015). In addition, the degree of satisfaction across all the parameters after the RBFDPs cemented at an increased OVD was reported high in this study, supported by the study of Creugers and De Kanter (2000) but they cemented the RBFDPs using a conformative approach. The present study reported no complications or failures of the RBFPDPs after 3 months. The findings are consistent with those of previous studies (Djemal et al., 1999; Lim et al., 2022a; Shahdad et al., 2018). In the authors' opinion, no failures in the present study may result in a high satisfaction of the participants toward the treatment. Nonetheless, high patient acceptance was reported by a previous study despite a 90% of survival rate with 24% occurrence of decemention (Botelho et al., 2014). This could be due to the nature of these two studies being different which later was evaluated retrospectively.

In the present study, the demographic and clinical factors were not significantly associated with all patient satisfaction parameters toward RBFDPs except for gender. Males were more aware of the avoidance of loading on the RBFDPs compared to females. This is the only parameter that influenced the satisfactory outcome. Possibly, males are more conscious of the risk of failure because they were reported to have higher maximum bite force than females (Roffie et al., 2020). Almost half of the patients avoided the load, which is similar to the findings in a retrospective study (Botelho et al., 2006). Nonetheless, Lim and Ariff (2020) found that the level of satisfaction of RBFDPs did not differ by gender.

The present study reflected a high patient satisfaction level toward RBFDPs cemented at an increased OVD after a short follow‐up period of 3 months. Complete occlusal contact and force reestablishment were achieved during that time. Therefore, possibly, the level of patient satisfaction with this short‐term result can be postulated to be similar to the long‐term outcome. The long‐term clinical evaluation of RBFDPs recorded a high overall satisfaction after 9.4 years. Nevertheless, the two‐unit cantilevered RBFDPs in that study include tooth preparations and cementation using a conformative approach (Botelho et al., 2014). Three participants achieved partial occlusal reestablishment in this study. This finding is consistent with Banerji et al. (2014). However, they reported that their masticatory functions were not affected by a reduced number of occlusal contacts, possibly their occlusal forces were restored after 12 weeks (Idris et al., 2022). The localized tooth wear participants demonstrated partial occlusal reestablishment after 10 years, but none of them were symptomatic, which is consistent with this study (Gulamali et al., 2011). Therefore, patient satisfaction should not be affected by this potential shortcoming. These participants will be reviewed regularly, and preparation of the antagonist will be considered in case of no or partial occlusal contact reestablishment occurs after RBFDP placement with the Dahl concept.

In the authors' opinion, the complete occlusal reestablishment contributed to the high satisfaction level in this study. The satisfaction outcome may be different in the early stage after the cementation of RBFDPs because of the abrupt occlusal changes that might affect function and speech (Gulati et al., 2016). Therefore, in the future, assessment of patient satisfaction at different times, studies with longer review periods, and larger sample sizes to document any possible patient‐reported outcomes including masticatory function, chewing efficacy, presence of pain, discomfort, and esthetics may be recommended (Cushing et al., 1986). This questionnaire was adopted because it allows evaluation of immediate patient‐reported outcomes after the RBFDPs cemented at an increased OVD. In addition, the questionnaire parameters were designed for the RBFDP. A questionnaire on Oral Health Impact Profile would be suggested to assess patients' oral health‐related quality of life in future studies.

Furthermore, the application of the Dahl concept in Prosthodontics is still a point of contention (Goldstein & Campbell, 2022), particularly in managing interocclusal space of single tooth restorations. It is hoped that the present study has answered the immediate patient‐reported outcome after the RBFDPs cemented at an increased OVD.

## 5 CONCLUSIONS

The study can conclude that patient satisfaction toward RBFDPs cemented at an increased OVD was high on all parameters during the 12‐week review visit. However, males are more aware of avoiding a load of prostheses compared with females. As clinicians, it is reasonable to consider RBFDP cemented at an increased OVD as a favorable alternative for patients to create the metal retainer space.

## AUTHOR CONTRIBUTIONS

Tong Wah Lim conceptualized, designed, coordinated the study, analyzed data, and reviewed and edited the manuscript. Rostam Iffendi Idris performed the bite force acquisition and analyzed and interpreted the data. Melati Mahmud participated in writing the original draft, reviewing, and editing. All authors approve the content of the manuscript and agree to be held accountable for the work.

## CONFLICT OF INTEREST STATEMENT

The authors declare no conflict of interest.

## Data Availability

The data that support the findings of this study are available from the corresponding author upon reasonable request.

## References

[cre2774-bib-0001] Abduo, J. , & Lyons, K. (2012). Clinical considerations for increasing occlusal vertical dimension: A review. Australian Dental Journal, 57(1), 2–10. 10.1111/j.1834-7819.2011.01640.x 22369551

[cre2774-bib-0002] Banerji, S. , Mehta, S. B. , Kamran, T. , Kalakonda, M. , & Millar, B. J. (2014). A multi‐centred clinical audit to describe the efficacy of direct supra‐coronal splinting—A minimally invasive approach to the management of cracked tooth syndrome. Journal of Dentistry, 42, 862–871. 10.1016/j.jdent.2014.02.017 24589848

[cre2774-bib-0003] Botelho, M. G. , Leung, K. C. M. , Ng, H. , & Chan, K. (2006). A retrospective clinical evaluation of two‐unit cantilevered resin‐bonded fixed partial dentures. The Journal of the American Dental Association, 137(6), 783–788. 10.14219/jada.archive.2006.0290 16803807

[cre2774-bib-0004] Botelho, M. G. , Ma, X. , Cheung, G. J. K. , Law, R. K. S. , Tai, M. T. C. , & Lam, W. Y. H. (2014). Long‐term clinical evaluation of 211 two‐unit cantilevered resin‐bonded fixed partial dentures. Journal of Dentistry, 42(7), 778–784. 10.1016/j.jdent.2014.02.004 24685984

[cre2774-bib-0005] Creugers, N. H. J. , & De Kanter, R. J. A. M. (2000). Patients' satisfaction in two long‐term clinical studies on resin‐bonded bridges. Journal of Oral Rehabilitation, 27(7), 602–607. 10.1046/j.1365-2842.2000.00553.x 10931253

[cre2774-bib-0006] Cushing, A. M. , Sheiham, A. , & Maizels, J. (1986). Developing socio‐dental indicators—The social impact of dental disease. Community Dental Health, 3(1), 3–17. 3516317

[cre2774-bib-0007] Dahl, B. L. , Krogstad, O. , & Karlsen, K. (1975). An alternative treatment in cases with advanced localized attrition. Journal of Oral Rehabilitation, 2(3), 209–214. 10.1111/j.1365-2842.1975.tb00914.x 1056978

[cre2774-bib-0008] Dayanik, S. (2016). Resin‐bonded bridges—Can we cement them ‘high’? Dental Update, 43(3), 243–253. 10.12968/denu.2016.43.3.243 27439271

[cre2774-bib-0009] Djemal, S. , Setchell, D. , King, P. , & Wickens, J. (1999). Long‐term survival characteristics of 832 resin‐retained bridges and splints provided in a post‐graduate teaching hospital between 1978 and 1993. Journal of Oral Rehabilitation, 26(4), 302–320. 10.1046/j.1365-2842.1999.00374.x 10232858

[cre2774-bib-0010] Goldstein, G. , & Campbell, S. (2022). The Dahl concept: Best evidence consensus statement. Journal of Prosthodontics, 31(3), 196–200. 10.1111/jopr.13441 34626153

[cre2774-bib-0011] Gough, M. B. , & Setchell, D. J. (1999). A retrospective study of 50 treatments using an appliance to produce localised occlusal space by relative axial tooth movemen. British Dental Journal, 187(3), 134–139. 10.1038/sj.bdj.4800223 10481364

[cre2774-bib-0012] Gulamali, A. B. , Hemmings, K. W. , Tredwin, C. J. , & Petrie, A. (2011). Survival analysis of composite Dahl restorations provided to manage localised anterior tooth wear (ten year follow‐up). British Dental Journal, 211(4), E9. 10.1038/sj.bdj.2011.683 21869770

[cre2774-bib-0013] Gulati, J. S. , Tabiat‐Pour, S. , Watkins, S. , & Banerjee, A. (2016). Resin‐bonded bridges—The problem or the solution? Part 1: Assessment and design. Dental Update, 43(6), 506–521. 10.12968/denu.2016.43.6.506 29148644

[cre2774-bib-0014] Hebel, K. , Gajjar, R. , & Hofstede, T. (2000). Single‐tooth replacement: Bridge vs. implant‐supported restoration. Journal of Canadian Dental Association, 66(8), 435–438. 11040527

[cre2774-bib-0015] Idris, R. I. , Shoji, Y. , & Lim, T. W. (2022). Occlusal force and occlusal contact reestablishment with resin‐bonded fixed partial dental prostheses using the Dahl concept: A clinical study. The Journal of Prosthetic Dentistry, 127(5), 737–743. 10.1016/j.prosdent.2020.11.035 33455729

[cre2774-bib-0016] Jepson, N. , Allen, F. , Moynihan, P. , Kelly, P. , & Thomason, M. (2003). Patient satisfaction following restoration of shortened mandibular dental arches in a randomized controlled trial. The International Journal of Prosthodontics, 16(4), 409–414. 12956497

[cre2774-bib-0017] Lam, W. Y. H. , Chan, R. S. T. , Li, K. Y. , Tang, K. T. , Lui, T. T. , & Botelho, M. G. (2019). Ten‐year clinical evaluation of posterior fixed‐movable resin‐bonded fixed partial dentures. Journal of Dentistry, 86, 118–125. 10.1016/j.jdent.2019.06.003 31181243

[cre2774-bib-0018] Lim, T. W. (2022). Creating space for a resin‐bonded fixed partial denture retainer by using the Dahl concept. Journal of Prosthetic Dentistry, S0022‐3913(22), 00227–0022. 10.1016/j.prosdent.2022.03.036 35577613

[cre2774-bib-0019] Lim, T. W. , Ab Ghani, S. M. , & Mahmud, M. (2022a). Occlusal re‐establishment and clinical complications of resin‐bonded fixed partial dental prostheses cemented at an increased occlusal vertical dimension: A retrospective study. Journal of Prosthetic Dentistry, 127(2), 258–265. 10.1016/j.prosdent.2020.06.034 33279159

[cre2774-bib-0020] Lim, T. W. , Ab Ghani, S. M. , & Mahmud, M. (2022b). Response to letter to editor on “occlusal re‐establishment and clinical complications of resin‐bonded fixed partial dental prostheses (RBFPDP) cemented at an increased occlusal vertical dimension (OVD)”. Journal of Prosthetic Dentistry, 127(4), 671–672. 10.1016/j.prosdent.2021.11.013 34924188

[cre2774-bib-0021] Lim, T. W. , Ab Ghani, S. M. , & Mustaza, T. A. (2014). Resin bonded bridges‐revisited. Malaysian Dental Journal, 36, 24–29.

[cre2774-bib-0022] Lim, T. W. , & Ariff, T. F. T. M. (2020). Single tooth implant versus resin‐bonded bridge: A study of patient's satisfaction. European Journal of General Dentistry, 9, 90–95. 10.4103/ejgd.ejgd_63_20

[cre2774-bib-0023] Malmstrom, H. , Dellanzo‐Savu, A. , Xiao, J. , Feng, C. , Jabeen, A. , Romero, M. , Huang, J. , Ren, Y. , & Yunker, M. A. (2015). Success, clinical performance and patient satisfaction of direct fibre‐reinforced composite fixed partial dentures—A two‐year clinical study. Journal of Oral Rehabilitation, 42(12), 906–913. 10.1111/joor.12327 26172283

[cre2774-bib-0024] Moreno‐Hay, I. , & Okeson, J. P. (2015). Does altering the occlusal vertical dimension produce temporomandibular disorders? A literature review. Journal of Oral Rehabilitation, 42(11), 875–882. 10.1111/joor.12326 26140528

[cre2774-bib-0025] Ormianer, Z. , & Gross, M. (1998). A 2‐year follow‐up of mandibular posture following an increase in occlusal vertical dimension beyond the clinical rest position with fixed restorations. Journal of Oral Rehabilitation, 25(11), 877–883. 10.1046/j.1365-2842.1998.00326.x 9846908

[cre2774-bib-0026] Poyser, N. J. , Porter, R. W. J. , Briggs, P. F. A. , Chana, H. S. , & Kelleher, M. G. D. (2005). The Dahl concept: Past, present and future. British Dental Journal, 198(11), 669–676. 10.1038/sj.bdj.4812371 15951771

[cre2774-bib-0027] Redman, C. D. J. , Hemmings, K. W. , & Good, J. A. (2003). The survival and clinical performance of resin based composite restorations used to treat localised anterior tooth wear. British Dental Journal, 194(10), 566–572. 10.1038/sj.bdj.4810209 12819732

[cre2774-bib-0028] Roffie, J. , Lim, T. W. , Patar, M. N. , Abdullah, S. A. , & Ghani, H. A. (2020). Wireless sensor network for a bite force recorder. Journal of International Dental and Medical Research, 13(4), 1592–1597.

[cre2774-bib-0029] Shahdad, S. , Cattell, M. J. , Cano‐Ruiz, J. , Gamble, E. , & Gambôa, A. (2018). Clinical evaluation of all ceramic zirconia framework resin bonded bridges. The European Journal of Prosthodontics and Restorative Dentistry, 26(4), 203–211. 10.1922/EJPRD_01810Shahdad09 30398816

[cre2774-bib-0030] Thoma, D. S. , Sailer, I. , Ioannidis, A. , Zwahlen, M. , Makarov, N. , & Pjetursson, B. E. (2017). A systematic review of the survival and complication rates of resin‐bonded fixed dental prostheses after a mean observation period of at least 5 years. Clinical Oral Implants Research, 28(11), 1421–1432. 10.1111/clr.13007 28191679

